# Economic impact of interventional study on rational use of antiseptics and disinfectants in Super Speciality Hospital of Nagpur

**DOI:** 10.4103/0253-7613.41043

**Published:** 2008

**Authors:** Vandana Agarwal, Kunda Gharpure, Vijay Thawani, Sushil Makhija, Anita Thakur, Rajaram Powar

**Affiliations:** Government Medical College, Nagpur - 440 003, India; 1 Super Speciality Hospital, Nagpur - 440 003, India

**Keywords:** Antiseptics, disinfectants, cost containment

## Abstract

**Objective:**

To measure the impact of interventions on rational use of antiseptics and disinfectants (A and D) for cost containment in Super Speciality Hospital (SSH) of Government Medical College, Nagpur (GMCN), India.

**Materials and Methods:**

This study was conducted from October 2003 to March 2007 in SSH of GMCN. In the pre-interventional phase (Phase-I), purchase, stocking and distribution of A and D was studied to find problem areas. Based on this formative data an intervention was planned (Phase-II) during which rationing of the A and D was done. Rational quantities needed for different A and D procedures were calculated based on recommendations of National Aids Control Organization (NACO) with modifications to suit our hospital setup. Detailed information, education, communication and training about rational use of A and D were provided to the hospital staff. In the post-interventional phase (Phase-III), the use of A and D was rationalized at the distribution level and the efficacy of in-use A and D was tested at user sites. Data about medicine expenditure, patient record and A and D usage in various departments was obtained from hospital records. Savings on A and D as against total annual medicine expenditure was calculated taking the cost of A and D in the post-intervention period.

**Results:**

The expenditure on A and D as a result of intervention decreased by 20.7%. Out of the total medicine expenditure, the expenditure on A and D which accounted for 6.2% before intervention, decreased to 1.95% after the intervention.

**Conclusion:**

The information, education and communication (IEC) interventions attempted by us resulted in significant decrease in the use and expenditure of A and D.

The health care industry has devoted relatively little technical talent and intellectual effort in optimizing its operations. This neglect has contributed to the development of a high-cost delivery system with poor performance and highly uneven reach of quality care.[1] The economic objective of a medicine supply system is to ensure provision of safe, effective and good quality medicines at minimum possible cost. Efficient supply management system must also ensure fewer stock shortages and fewer losses of medicines.[2] Value analysis and cost reduction are common features of enhancing product value by improving the relationship of worth to cost and then taking steps to perform the same function cheaply.[3] Cost reduction represents real reduction in unit cost of services rendered without impairing the suitability in the use for which it is intended. This is brought about by elimination of wasteful elements and at the same time retaining its essential characteristics and quality. Essential features of cost reduction are planned approach, co-operation and team work. Whereas cost control is a routine exercise to improve operational efficiency, cost reduction program is a planned approach, which is based on co-operation, at all levels, operational and procedural research and proper follow-up. Techniques used to attain cost reduction are value analysis, standardization and rationalization.[3] This economic principle when applied to hospital inventory takes into consideration the reduced cost of medicines through procurement, supply chain efficiencies, human resource management, inventory control, and disease burden. However, studies on rational use of antiseptics and disinfectants (A and D) and the resultant cost containment are not available in literature.

Super Speciality Hospital (SSH) is an autonomous 120-bedded hospital of Government Medical College, Nagpur. Started in 1995 by the Government of Maharashtra to provide specialized care in cardiology, nephrology, neurology and gastroenterology, it has its own medical store with independent medicine purchase and distribution system. During the economic analysis, done as a part of managerial exercise, it was noticed that the annual expenditure on A and D in SSH was increasing which was disproportionate to the increase in patient attendance and services. The issue was discussed with SSH Infection Control Team (ICT), whose surveillance report revealed that the concentrations and indications for use of A and D were not as per the standard recommendations.[4] These deficiencies coupled with lack of data on rational use of A and D and resultant cost burden prompted us to undertake this study adopting rationalization of A and D as an intervention for cost reduction and measurement of the outcome in economic terms vis-à-vis the annual total medicine expenditure.

## Materials and Methods

This study was conducted from October 2003 to March 2007. It was divided into three phases with additional follow-up.

### Phase I - Pre-interventional phase

(October 2003 to September 2004): It comprised of analyzing the purchase, stocking and distribution of A and D. During this phase, the distribution policy for A and D was to supply what was demanded by the users.

### Phase II - Interventional phase

(October 2004 to September 2005): It comprised of information, education and communication (IEC) measures like preparing and disseminating recommendations for rational use of A and D and conducting training for the end users for proper use of A and D. During this phase the distribution of A and D was rationed. The intervention was executed in three steps:

### Step I: IEC and training to rationalize the use of A and D:

The ICT staff during weekly rounds imparted teaching on rational use of A and D to medical and paramedical staff. Recommendations for rational use of A and D based on the guidelines of National AIDS Control Organization (NACO), India[4] were prepared and handouts distributed liberally to all departments, wards, operation rooms and also pasted at all workstations of the users for ready reference (Annexure 1). Guidelines for disinfection and sterilization of hospital articles were prepared as per the recommendations of NACO. Handouts of these were distributed to all hospital staff, including nursing staff for implementation (Annexure 2). Interactive demonstrations were organized once a month for the nursing staff to update their knowledge, attitude and practice (KAP). Interactive training for the attendants and sanitary workers was conducted for re-enforcement of education. Handouts were distributed to sanitary workers in local vernacular language and questions encouraged from them. A mobile workshop on wheels was conducted during which the correct use of A and D for disinfecting articles was shown. Demonstrations were given on preparation of correct concentrations of A and D and disinfection of hospital waste prior to disposal.

**Annexure 1:** Rational use of antiseptics and disinfectants

*Name*	*Recommended for*	*Working concentration*
Benzalkonium chloride		
a) Saniquad - P 20% W/V	a) for hand wash	For Saniquad - P*
b) Benzotate 40% V/V	b) for cheatle forceps	a) 30 ml in 3 liters of water (1%)
	c) cleaning endoscope, catheters lab instruments before disinfection	b) 8 ml in 400 ml of water (2%)
		c) 20 ml in 1 liter of water (2%)
Iodine		
a) Povidone iodine PI scrub 7.5% W/V	a) scrub for surgical hand wash	a) use undiluted PI scrub
b) Povidone iodine solution 5% W/V	b) painting of skin	b) use undiluted PI scrub
	c) dressing	c) use undiluted PI scrub
Sodium hypochlorite		
a) Aquaclore 5%	a) swabbing of floor	a) 10 ml in 1 liter of water (1%)
	b) disinfecting all laboratory	b) 20 ml in 1 liter of water (2%)
	Glassware, needles, syringes, Gloves, tissues etc.	c) 20 ml in 1 liter of water (2%)
	c) disinfecting spills	
Black coal tar disinfectant,		
a) Phenyl,	Only for disinfecting toilets	scrub toilet with detergent
b) Liquophin	Do not use any other disinfectant/antiseptic like	soap. Wash with phenyl water
	Aquaclore/Saniquad - P	(50 ml phenyl in 10 liter of water)
Gluteraldehyde		
a) Glutasol 2% W/V	Sterilization of instruments which are destroyed by heat like endoscope	Add activator to 5 liters and use this for 14 days
Hydrogen peroxide 6%	a) dressing of infected wounds	a) use undiluted
	b) cleaning of tubes and catheters to remove blood clots	b) use undiluted
Denatured spirit	a) disinfection of furniture (tables, chairs etc.)	a) add 30 ml water to 70 ml spirit
	b) as antiseptic at injection site	b) add 30 ml water to 70 ml spirit

* For Benzotate half volume of Saniquad - P; For any other disinfectant purpose, use only detergent powder

**Annexure 2:** Recommendations for sterilization and disinfection of hospital articles

*Hospital articles*	*Disinfectant concentration*	*Procedure*
Used gloves, syringes, needles	Sodium hypochlorite 2%	Immerse for 30 min. Disinfectant to be changed 3 times daily
Cheatle forceps, thermometer	Benzalkonium chloride 2%	To be changed daily
Endoscopy instruments, suction tubes, all catheters, ventilator units, endo-tracheal tubes, laryngoscope, ambu bags	Glutaraldehyde 2%	Immerse for 30 min. Rinse 3-4 times with sterile water. Gluteraldehyde to be reused upto14 days
Suction bottle	Sodium hypochlorite 2%	Immerse for 30 min. Wash with soap and water. Autoclave
Removal of blood clots from tubes and catheters	Hydrogen peroxide 6%	Immerse for 15 min. Wash with soap and water. Autoclave.
Oxygen mask, radiant warmer phototherapy machine	Denatured spirit 70%	Wipe with soft linen thoroughly
Soiled linen	Sodium hypochlorite 1%	Immerse for 30 min. Dry and send to laundry
Mattresses, pillows, blankets, trolleys, cots and furniture	Denatured spirit 70%	a) Clean the waterproof plastic cover with spirit. Expose to formalin vapors (fumigation)
		b) Clean with detergent. Wipe with disinfectant
Bed pans, urine pots	Detergent 1%	Wash with hot water and immerse in 1% detergent solution. Rinse with water
Blood spills on floor	Sodium hypochlorite 2%	Pour disinfectant over the spill cover with absorbent cotton for 30 min. Discard. Mop floor with water
Floor washing	Sodium hypochlorite 1%	Scrub floor with detergent. Mop with disinfectant

### Step 2: Monitoring the efficacy of in-use disinfectants:

The in-use test was performed during phase II and III at monthly intervals to confirm that the chosen disinfectants viz. benzalkonium chloride, glutaraldehyde and sodium hypochlorite were effective under the actual in-use conditions, in recommended concentrations and for the recommended period of use. The test was performed on diluted disinfectants after they were used and left overnight as per the recommended procedure.[5] During this 1 ml of the disinfectant was diluted in 9 ml of nutrient broth and mixed well. Then 0.02 ml of the mixture was spot inoculated onto ten different areas of each of the two nutrient agar plates. One plate was incubated at 37°C for three days and the other plate was incubated at room temperature for seven days. The test was read as showing failure of disinfectant if there was growth in more than five areas on either plate.

### Step 3: Formulation of rational policy for A and D distribution:

The quantity of various A and D to be distributed monthly by the SSH medical store was calculated on the basis of

1. Indication for use as per NACO recommendations
2. Working concentration as per NACO recommendation
3. Manufacturers guidelines
4. Clinical need based on patient attendance
5. Number of procedures/investigations performed
6. The floor space area.

Information about the floor space was procured from Public Works Department, Government of Maharashtra of SSH Nagpur. The distribution quantity was calculated by adding 10% excess allowance to the quantity required for each A and D and this figure was then rounded to the nearest unit pack size (Annexure 3). To check the possible pilferage, the policy of taking empty containers back before issuing the next indent was started.

**Annexure 3:** Rationalized monthly distribution policy of antiseptics and disinfectants in Super Speciality Hospital, Nagpur

	*Number of units of antiseptics and disinfectants*						
	
	*B*	*G*	*H*	*HP*	*I*	*P*	*S*
Unit volume	540 ml	5 L	5 L	500 ml	500 ml	5 L	1 L
Nephrology	2	NIL	5	10	12	5	10
Gastroenterology	4	2	3	NIL	1	3	3
Neurosurgery	2	NIL	3	NIL	5	4	10
CVTS	2	NIL	3	2	10	4	10
Cardiology	2	6	5	10	15	6	13
Biochemistry	4	NIL	1	NIL	1/4 mth	1	4
Blood bank	1	NIL	1	NIL	NIL	1	2
Pathology	NIL	NIL	2	1	NIL	1	4
Microbiology	NIL	NIL	1	NIL	NIL	NIL	2
Radiology	1/4 mth	NIL	1/2 mth	NIL	NIL	3/4 mth	1/4 mth
Outpatient department	6	NIL	1	NIL	NIL	2	1
Operation theatre	1	4	7	2	35	1	20
General hospital	NIL		10	NIL	NIL	5	NIL

B = benzalkonium chloride, G = glutaraldehyde, H = sodium hypochlorite, HP = hydrogen peroxide, I = iodine, P = phenyl, S = denatured spirit

### Phase III - Post interventional phase

(October 2005 to September 2006): During this phase the distribution policy of A and D was strictly adhered to as per the quantity calculated in step 3 of phase II.

### Follow-up

(October 2006 to March 2007): The impact of the intervention was reassessed after six months of completion of phase III for sustenance of effect.

#### Data collection

The patient related data was obtained from the medical record section of SSH. Computerized data on annual patient attendance and number of annual procedures and investigations during each phase of the study was retrieved from respective sections. The A and D related computerized data was obtained from the medical store of SSH. Seven A and D were on hospital purchase list viz. benzalkonium chloride (B), glutaraldehyde (G), sodium hypochlorite (H), hydrogen peroxide (HP), iodine (I), phenyl (P), and denatured spirit (S). The annual unit distribution, average cost per unit and annual expenditure was calculated for each A and D during each phase of the study.

## Results

The floor space areas in square meters were found to be 938.3 for each in-patient department, 1220.2 for operation theatre complex, 382.3 each for blood bank and Biochemistry dept, 503.8 for Pathology and Microbiology departments combined, 299.7 for Radiology and 785.4 for common areas (porch, corridors, stair case and administrative block). The actual area in use showed minor variation, which was considered in calculating the effective floor area for each department. The effective in-use floor area remained constant for the entire study period.

Table 1 shows the turnover of patients and services in SSH. There is a noticeable increase in the annual patient attendance from phase I to phase III. A marked increase in patient service utilization was seen in Cardiology, CVTS, Microbiology, Blood bank and Biochemistry department because of introduction of some newer procedures and investigations, while others did not show much variance. Comparison of patient attendance and procedure-load in phase I and phase III showed significant increase (*P* = 0.0448).

**Table 1:** Patient attendance, procedures and investigations performed in Super Speciality Hospital, Nagpur

	*Phase I*	*Phase II*	*Phase III*
Annual patient attendance			
OPD	64983	80648	88710
Radiology	4545	5255	5298
Annual procedures			
Nephrology	2631	2681	2160
Gastroenterology	2173	3198	3094
Neurosurgery	509	482	499
CVTS	139	319	345
Cardiology	9415	13269	16768
Annual investigations			
Biochemistry	59349	64320	72706
Blood bank	7068	8056	9536
Pathology	18053	20554	20102
Microbiology	479	3567	6791

Mean - 5151, SEM - 2247, T value - 2.29, Degree of freedom - 10, ‘*P*’ value - 0.0448  by paired ‘t’ test using Graph pad Instat version 3.02 when phase I compared with phase III

Table 2 shows that there is a progressive decline in the annual unit consumption of A and D from phase I to phase III. Maximum decrease (174 units) was seen in the use of hydrogen peroxide by Nephrology department. Maximum increase (63 units) was seen in use of denatured spirit in operation rooms. In phase I, there was probably maximum irrational use of A and D by Nephrology, followed by Gastroenterology and Cardiology departments. This is evident from the decrease in all A and D after implementation of the rational policy in phase III. The decrease in use was statistically significant for B, H and P (*P* < 0.05).

**Table 2:** Effect of intervention on unit consumption of antiseptic and disinfectants

*Antiseptics and disinfectants*	*Benzalkonium chloride*		*Gluteraldehyde*		*Hydrogen Peroxide*		*Iodine*		*Denatured Spirit*		*Sodium Hypochlorite*		*Phenyl*	

*Unit volume*	*540 ml*		*5L*		*500 ml*		*500 ml*		*1 L*		*5L*		*5L*	

*Departments*	*Phase I*	*Phase III*	*Phase I*	*Phase III*	*Phase I*	*Phase III*	*Phase I*	*Phase III*	*Phase I*	*Phase III*	*Phase I*	*Phase III*	*Phase I*	*Phase III*
Nephrology	156	57	7	0	212	38	99	33	117	100	79	75	100	52
Gastroenterology	86	38	10	5	2	1	10	11	93	44	120	25	49	26
Neurosurgery	42	24	6	0	9	2	71	37	70	61.5	65	44	12	18
CVTS	13	12	2	0	10	2	24	26	28	48	63	20	33	19
Cardiology	94	54	62	42	31	13	84	54	155	88	138	66	41	25
Biochemistry	55	23	0	0	0	0	0	0	45	22	8	12	10	5
Blood bank	22	10	0	0	0	0	0	0	15	12.5	18	12	9	11
Pathology	15	2	0	0	0	0	0	0	10	24	16	8	8	9
Microbiology	0	0	0	0	0	0	0	0	0	0	0	4	0	0
Radiology	2	5	0	0	0	0	2	0	5	0	13	8	11	4
OPD	5	40	0	0	0	0	0	1	2	1	41	24	37	23
OT	114	41	14	16	12	10	158	219	85	148	97	61	7	12
General Hospital	8	0	0	0	0	0	0	0	0	0	122	110	58	35
Mean	23.538		2.923		16.154		5.1538		5.8462		23.923		10.462	
SEM	9.7373		1.6151		13.235		7.925		8.7461		8.382		4.1812	
T value	2.42		1.81		1.22		0.65		0.67		2.85		2.5	
Degree of freedom	12		12		12		12		12		12		12	
‘*P*’ value	0.0325		0.0954		0.2457		0.5277		0.5165		0.0145		0.0278	

B = benzalkonium chloride, G = glutaraldehyde, H = sodium hypochlorite, HP = hydrogen peroxide, I = iodine, P = phenyl, S = denatured spirit. *P* value by paired ‘t’ test using Graph pad Instat version 3.02

The decline in unit consumption was reflected in the decrease in expenditure of A and D in spite of the inflationary increase in unit cost [Table 3]. The monetary saving was calculated taking the cost of the individual A and D for the year in phase III. In phase I the expenditure was INR 2,67,188 while in phase III it came down to INR 2,11,891 (*P* < 0.05). The total saving thus amounted to INR 55, 297 which was 20.69%.

**Table 3:** Effect of intervention on expenditure of antiseptic and disinfectants

	*Expenditure in INR Phase I*	*Expenditure in INR Phase III*
Antiseptic		
Benzalkonium Chloride	36720	19890
Gluteraldehyde	35222	23047
Sodium hypochlorite	64701	59938
Hydrogen peroxide	3698	924
Iodine	27144	25196
Phenyl	74284	54322
Denatured spirit	25419	28574
total	267188	211891
% saving	20.69	
Statistics		
Mean	7899.6	
SEM	3226.6	
T Value	2.45	
Degree of freedom	6	
‘*P*’ value	0.0499	

INR = Indian National Rupees, Paired ‘t’ test was applied using Graph pad Instat version 3.02

Table 4 shows the total medicine expenditure for consecutive financial years from 2001 to 2007 and the corresponding expenditure on A and D. It can be appreciated that the intervention has been able to check the expenditure of A and D considerably. The expenditure on A and D out of the total medicine expenditure in the years before intervention was progressively on the rise (3.4%, 4.8%, 6.2%) which came down to 3.8% and then to 2.7% after intervention. It was found that for 2005-06, the total medicine expenditure was paradoxically less (21.64 lacs) because of non-supply of medicines from the regular suppliers, while A and D stock was maintained as found by us from the stock out periods. Hence, the percentage spent on A and D in that year is higher at 7% of the total medicine expenditure.

**Table 4:** Expenditure on antiseptics and disinfectants as percentage of total medicine expenditure

*Year*	*Total medicine expenditure*	*A + D expenditure (calculated for financial year)*	*% of total medicine expenditure*
2001	8880317	301947	3.4
2002	6382336	306707	4.8
2003	5240022	324570	6.2
2004	6218657	238725	3.8
2005	2164088	152528	7.0*
2006	5967416	162337	2.7**

All figures in INR

* Apparent expenditure (see text for details)

** *‘P’* = 0.0392 after regression analysis

Ours being a super speciality hospital, the patient intake and procedures performed can never be consistent because the procedures and investigations are very specific to the patients. This is evident from the variable figures of annual medicine expenditure.

The in-use test showed that for each of the three disinfectants viz. benzalkonium chloride, glutaraldehyde and sodium hypochlorite, the organism load was within the permissible limits on all occasions, which confirmed that the concentrations and procedures recommended by us for use of A and D were effective.

**Figure 1:**
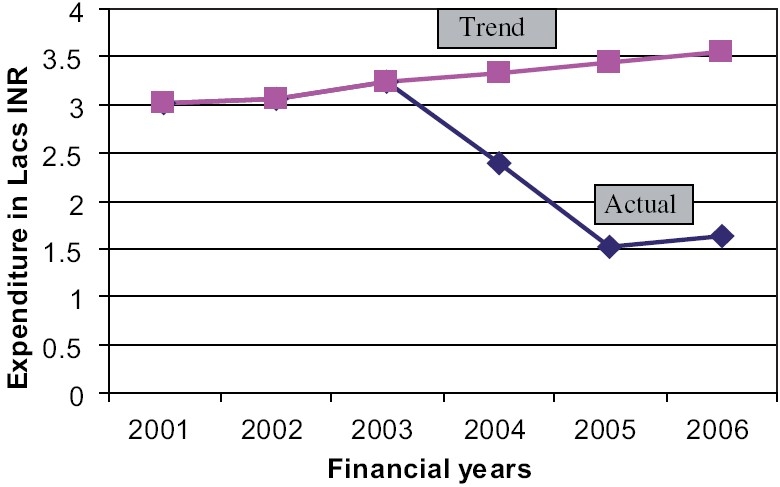
Trend and actual expenditure of antiseptics and disinfectants in Super Speciality Hospital, Nagpur

Figure 1 shows that during 2001-03 the annual expenditure on A and D was steadily rising, and suddenly dropped after the intervention, indicating the effect of interventions. If the 2001-03 data is extrapolated to get the future trend line, in the absence of any intervention, it shows a linear rise. Because of fluctuating annual patient population the forecast could not be done for total expenditure on all medicines.

## Discussion

**Rational use** - Rationalizing use of available resources leads to better management enabling distribution system to work efficiently. In our study rationalizing the use of A and D led to a noticeable decline in the utilization of the A and D. Involving all user cadres in implementing the policy had a definite advantage. From the economic aspect, this study gives us reason to proclaim that the IEC intervention has succeeded in cost reduction. It was observed that the quantity of A and D calculated for issuing to the user facilities was on the higher side and the demand from them was within this permitted quantity. In some cases like the increase in use of spirit in OT and OPD, this was due to increased awareness about its proper utilization, which was perhaps not there earlier.

**Efficacy** - In interventional as well as post-interventional phases, monitoring the efficacy of A and D was done to ensure that the purpose of the disinfectant was not lost. In spite of the decrease in the quantity of A and D issued to almost all departments, the test of in-use A and D was a success. It shows that the A and D available on SSH purchase list were capable of taking care of all the disinfection and antiseptic procedures in the recommended dilutions.

**Decrease in wastage** - Through the implementation of the intervention, it was possible to check the irrational use of A and D thus decreasing the wastage, paving way for optimal utilization of the resources.

**Pilferage** - The policy of issuing of fresh supplies against return of empty containers was deterrent to pilferage of A and D.

**The outlier** - The annual medicine expenditure for the year 2005-2006 was far below the average expenditure of the previous five years. This was probed into and it was found that the medicine purchase orders worth INR 47.32 lakhs were placed with regular suppliers but due to reasons beyond the control of medical stores, the supply of medicines was poor, causing many medicine stock outs. However, for A and D under study, out of seven, four were available throughout this period. Therefore, during this period the expenditure on A and D out of total medicine expenditure was disproportionately high (7%). This was also due to the fact that in absence of supplies from regular suppliers, purchases of A and D were made at a higher price from local market to cater to the hospital needs.

### Regression analysis:

The expenditure on A and D in pre intervention years showed a typical rising trend. Extrapolating these values for our study period using regression line for further four years, showed clearly the expected rise. As against this, the actual expenditure decreased throughout the study years and the effect of interventions continued for the next six-month period showing a sustained effect.

A study on material management system in public health care has identified areas for possible time and cost reduction.[6] Ways of cost containment in hospitals have been suggested in another study.[7] Material management has taken over the health sector so extensively that professional agencies provide services for cost containment programmes.[8,9] A management group claims to have reduced cost of medicines used in primary care by 60% of retail price through procurement and supply chain efficiencies. Similarly, hypertension treatment cost was reduced by 80% to INR 10 per month through scientific classification of patients based on disease burden.[8]

## Conclusion

In a super speciality hospital like ours with fluctuating patient intake, investigations and procedures it is difficult to allocate any budgetary provisions in advance, based on forecasting methodology. In spite of that, without compromising on the efficacy of A and D, it is beyond question that our IEC intervention resulted in a marked decrease in expenditure of A and D.
